# Membrane Lipidome Reorganization and Accumulation of Tissue DNA Lesions in Tumor-Bearing Mice: An Exploratory Study

**DOI:** 10.3390/cancers11040480

**Published:** 2019-04-04

**Authors:** Marios G. Krokidis, Maria Louka, Eleni K. Efthimiadou, Sevasti-Kiriaki Zervou, Kyriakos Papadopoulos, Anastasia Hiskia, Carla Ferreri, Chryssostomos Chatgilialoglu

**Affiliations:** 1 Institute of Nanoscience and Nanotechnology, N.C.S.R. “Demokritos”, 15310 Agia Paraskevi-Athens, Greece; m.krokidis@inn.demokritos.gr (M.G.K.); e.efthimiadou@inn.demokritos.gr (E.K.E.); s.zervou@inn.demokritos.gr (S.-K.Z.); k.papadopoulos@inn.demokritos.gr (K.P.); a.hiskia@inn.demokritos.gr (A.H.); 2 Lipidomics Laboratory, Lipinutragen srl, Via Piero Gobetti 101, 40129 Bologna, Italy; mar_louka@yahoo.gr (M.L.); carla.ferreri@isof.cnr.it (C.F.); 3 Department of Chemistry, National and Kapodistrian University of Athens, 15784 Zografou, Greece; 4 ISOF, Consiglio Nazionale delle Ricerche, Via Piero Gobetti 101, 40129 Bologna, Italy

**Keywords:** tumor-bearing mice, fatty acid-based lipidomics, membrane phospholipids, oxidative lesions, genotoxic stress

## Abstract

Increased rates of reactive oxygen/nitrogen species (ROS/RNS) are involved in almost all cancer types, associated with tumor development and progression, causing damage to biomolecules such as proteins, nucleic acids and membrane lipids, in different biological compartments. We used a human tumor xenograft mouse model to evaluate for the first time in parallel the remodeling of fatty acid moieties in erythrocyte membrane phospholipids and the level of ROS-induced DNA lesions in liver and kidney tissues. Using liquid chromatography tandem mass spectrometry the 5′*R* and 5′*S* diastereoisomers of 5′,8-cyclo-2′-deoxyadenosine and 5′,8-cyclo-2′-deoxyguanosine, together with 8-oxo-7,8-dihydro-2′-deoxyadenosine, were determined in mice at young (4- and 5-weeks) and old (17-weeks) ages and compared with control SCID mice without tumor implantation. Tumor-bearing mice showed a higher level of ROS-damaged nucleosides in genomic DNA as the age and tumor progress, compared to controls (1.07–1.53-fold in liver and 1.1–1.4-fold in kidney, respectively). The parallel fatty acid profile of erythrocyte membranes showed a profound lipid remodeling during tumor and age progression consisting of PUFA consumption and SFA enrichment (ca 28% and 58%, respectively, in late stage tumor-bearing mice), markers of enhanced oxidative and proliferative processes, respectively. Membrane lipid remodeling and ROS-induced DNA lesions may be combined to afford an integrated scenario of cancer progression and ageing, reinforcing a holistic vision among molecular markers rather than the biomarker identification in a single compartment.

## 1. Introduction

The overall consequences of free radical and oxidative processes, generated from physiological cellular metabolism or external sources and involved in disease and aging processes, have been examined in the last decades with attention to the balance between antioxidant protection and the signaling role covered by the oxidation products for adaptive or apoptosis programs [1,2,3]. The fate of a living organism in developing pathologies, including age-related degenerative diseases and cancer, is also connected with inflammatory responses that play decisive roles at different stages of these diseases [4]. In this complex scenario the possibility to obtain parallel information from known biomarkers of nucleic acids and membrane lipid transformations is attractive, being both essential molecular elements for cell formation and playing key roles in tumorigenesis.

The oxidatively-induced nucleoside modifications, like 5′,8-cyclo-2′-deoxyadenosine (cdA) and 5′,8-cyclo-2′-deoxyguanosine (cdG), are well recognized products of nucleic acid damage [5,6]. Both cdA and cdG, existing as 5′*R* and 5′*S* diastereoisomeric forms, are generated by the reaction of hydroxyl radicals (HO^•^) with the genetic material via C5′ radical chemistry of purine moieties (cf. Figure S1 in Supplementary Materials for structural details) [7,8,9]; they are considered the “smallest” tandem lesions and they are substrates of nucleotide excision repair (NER). These lesions cannot be removed by the base excision repair (BER) system, unlike 8-oxo-7,8-dihydro-2′-deoxyadenosine (8-oxo-dA) lesion, since the presence of the C5′–C8 covalent bond between the sugar and the base moieties prevents from the cleavage of the glycosidic bond by DNA glycosylase [10,11]. Several features of the purine 5′,8-cyclo-2′-deoxynucleoside (cPu) lesions render them interesting for follow-up of diseases: (i) the structural features for the recognition of the four cPu lesions by the human NER, as determined in human HeLa cell extracts, showed that cdA and cdG lesions are excised with similar efficiencies, but the 5′*R*-diastereoisomers of both cdA and cdG are better substrates of NER than the corresponding 5′*S* ones [12]; (ii) when these lesions are inefficiently removed, they lead to blockage of DNA replication, weak lesion bypass DNA synthesis by translesion DNA polymerases, RNA polymerase II stalling and transcriptional mutagenesis [10,11,13,14]. For example, a cdA lesion located in a trinucleotide repeat CAG tract can be bypassed directly by pol β, skipping over the lesions, thereby resulting in CTG repeat deletion [15]; (iii) cdA bypass by pol β during DNA replication and BER can induce mutations and single-strand DNA breaks, leading to genome instability [16]. To further highlight the significant value of the cPu lesions, it is worth noting that their elevated levels in human cell lines and fluids as 5′*R* and 5′*S* diastereoisomers are associated with pathological processes, including neurological disorders, ageing and carcinogenesis [17,18,19,20]. Quantification of the four cPu lesions of DNA can be performed simultaneously [21], as shown recently in two distinct human breast cancer cell lines before and after exposure to radiation-induced DNA-damage conditions [22]. Oxidatively-induced DNA lesions accumulate in animal tissues [23] and cPu lesions are associated with aging processes, since their levels increase in liver, kidney and brain tissues of old wild-type and DNA repair deficient progeroid ERCC^−/Δ^ mice [24]. cdA and cdG accumulate in tissues of LEA and LEC rats, being implicated in transition metal-induced diseases such as Wilson’s disease [25,26], and their levels were reported in liver, spleen and brain tissues of prdx1^+/+^ and prdx1^−/−^ mice [27].

Apart from DNA damage, another important molecular pool, connected with cell growth and radical stress, as well as necessary to tumor replication, is the lipid composition of cell membrane [28,29,30]. Cancer development is characterized by a distinct lipid and, in particular, fatty acid metabolism [31,32]. It is well known that the transformation of membrane lipid composition affects its fluidity, permeability as well as membrane lipid-related signaling, and can give favorable signals for tumor initiation, progression and metastasis [33,34,35]. Membrane remodeling is affected by the intracellular lipid pool, which depends on both the endogenous fatty acid biosynthesis and the dietary intake, especially for the essential polyunsaturated fatty acid (PUFA) supply. Thus, membrane fatty acid composition is influenced by a combination of stabilized nutritional conditions and metabolic status [28]. As a matter of fact, tumor initiation and propagation are characterized by an altered activity of enzymes involved in lipid biosynthesis, such as fatty acid synthase (FASN) and desaturases. Indeed, together with the corresponding fatty acids, the increased enzymatic activities are pointed as significant markers of tumor presence and growth [32,34,36,37]. Membrane lipidomics can follow fatty acid changes with the advantage of giving additional and fundamental information on signaling lipids, based on the presence of ω-6 and ω-3 PUFA in cell membrane. The release of ω-6 PUFA gives rise to signaling cascades connected to inflammation or resolution, as well as to cell proliferation and apoptosis [38,39]. Moreover, the membrane-targeted stimuli and the oxidative damage to the lipid pool influence the membrane remodeling process, known as Lands’s cycle [40], influencing phospholipid turnover and their fatty acid content. This is an important molecular aspect to be followed up on an animal model before and during tumor progression, taking into account that lipid metabolism is a crucial step for proliferation and invasion [41]. Membrane remodeling is also considered in the ageing process, since it depends on enhanced oxidative consumption of the PUFA moieties in tissues. Erythrocyte membrane fatty acid levels can indicate the extent of exposure of a living organism, since this circulating cell type can be representative of the lipid content in tissues [39]. The membrane molecular status, influencing biophysical and chemical properties, such as fluidity, permeability and oxidizability, is well expressed by membrane indices calculated from the fatty acid families and types [29]. Moreover, free radical reactivity can also induce the transformation of the naturally occurring fatty acid cis double bonds into the most thermodynamically stable, but unnatural, geometrical trans isomer [42]. The follow-up of PUFA moieties of phospholipids can monitor such an endogenous transformation in cell membranes, and monotrans PUFA isomers are specific markers of this “geometrical” radical stress (cf. Figure S2 in Supplementary Materials for structural details), exclusively formed by thiyl radical-catalyzed processes [42].

Based on these premises, we thought to combine fatty acid-based membrane lipidomics with DNA damage information. We used an experimental diseased animal model, that is a severe combined immunodeficient (SCID) female mouse, inoculated with a human tumor cell line (human glioblastoma U87MG) and monitored at different ages (4- and 17-weeks). Such human xenografts are already known in preclinical research for assessment of anticancer therapeutics [43,44]. Identification and quantification of DNA lesions were obtained by isotope dilution LC-ESI-MS/MS analysis in two distinct tissues types (liver and kidney) [45,46]. In parallel we assessed fatty acid levels of the erythrocyte membrane for the same animals at the two ages. We anticipate the unprecedented observation of in vivo differences of oxidatively-induced DNA lesions in tissues and erythrocyte membrane lipids, and hypothesized possible connections between these two molecular pools in their response to both ageing and disease conditions.

## 2. Results

### 2.1. Protocol Outline

**Figure 1:**
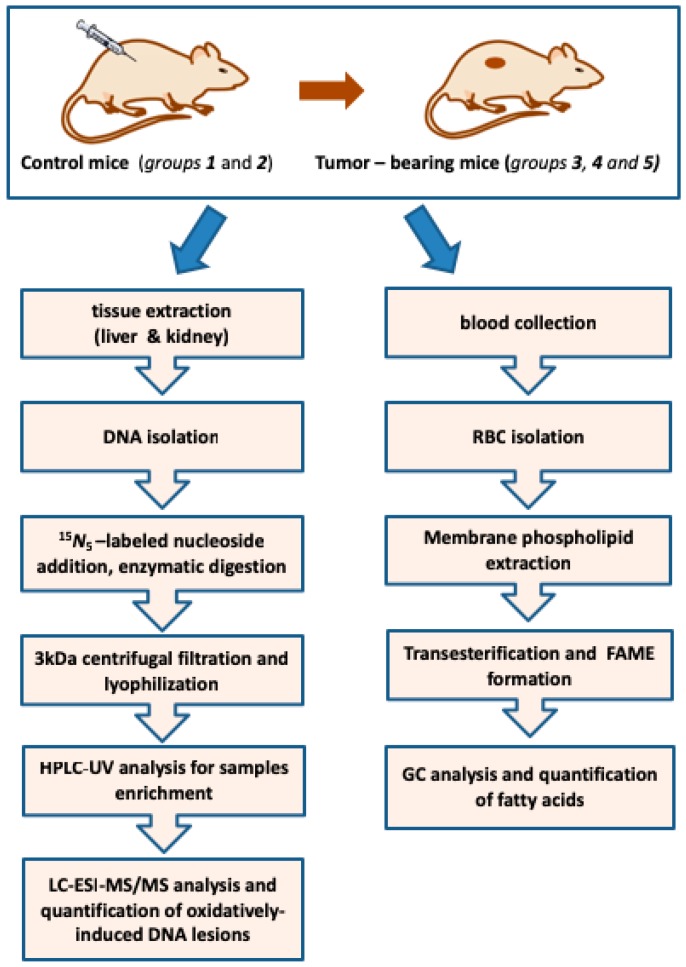
Flow chart for parallel analysis and quantification of oxidatively-induced DNA lesions in tissues and membrane fatty acid moieties in erythrocytes of SCID mice; *Group 1*: 4-weeks old; *Group 2*: 17-weeks old; *Group 3*: 4-weeks old; *Group 4*: 5-weeks old; *Group 5*: 17-weeks old.

Figure 1 provides a flow chart of the protocols used in our study. Human tumor xenografts are obtained by inoculating U87MG human brain glioblastoma cells subcutaneously in two-weeks-old SCID mice. Approximately 2 weeks post-injection, the first set of animals was sacrificed (*Group 3*, 4-weeks-old). The second set of tumor-bearing mice was sacrificed one week later (*Group 4*, 5-weeks-old) and the third set after 84 days (*Group 5*, 17-weeks-old). The *Group 3* and *Group 4* are referred as the early stage of tumorigenesis in our study, while the *Group 5* as the final stage of tumor presence, when all tumor-bearing mice are characterized by very poor conditions. In our study, we evaluated the same DNA lesions and fatty acids of membrane phospholipids in control SCID mice without tumor implantation, four weeks old (*Group 1*) and seventeen weeks old (*Group 2*), to identify potential differences with tumor-bearing animals. Since in our study human xenograft are selected and tumors originate by exogenous inoculation with human cancer cells, tumor tissues were not analyzed, following an approach described for a genetically engineered mouse [44]. Tumor causes alterations throughout the body, with some of the consequences comparable to chronic inflammation [47]. In carcinogenic processes, polymorphisms of DNA repair genes influence DNA repair systems and consequently oxidatively-induced DNA nucleosides are detected at elevated levels [48,49,50,51].

Genomic DNA was isolated from liver and kidney, hydrolyzed to single nucleosides by an enzymatic cocktail containing nucleases, and analyzed by liquid chromatography with tandem mass spectrometry for the determination of the modified nucleosides (the four cPu and 8-oxo-dA), as shown in Figure 1, in accordance to a recently optimized protocol [21,22]. The fatty acid-based membrane lipidomic study was performed using membrane phospholipids isolated from red blood cells (RBCs) with known procedures [52]. As shown in Figure 1, the extracted RBC phospholipids were transformed to fatty acid methyl esters (FAMEs) under reported conditions [53]. FAMEs were analyzed by gas chromatography (GC) in order to identify saturated (SFA) and unsaturated (MUFA and PUFA) fatty acids, the latter being recognized as positional and geometrical isomers [54]. Comparison of healthy Swiss and SCID mice was also performed. Although it is not the scope of this article to focus on the differences between these two mice models, some important information is obtained related to immuno-deficiency.

### 2.2. Oxidatively-Induced DNA Lesions Accumulation in Organs

**Figure 2:**
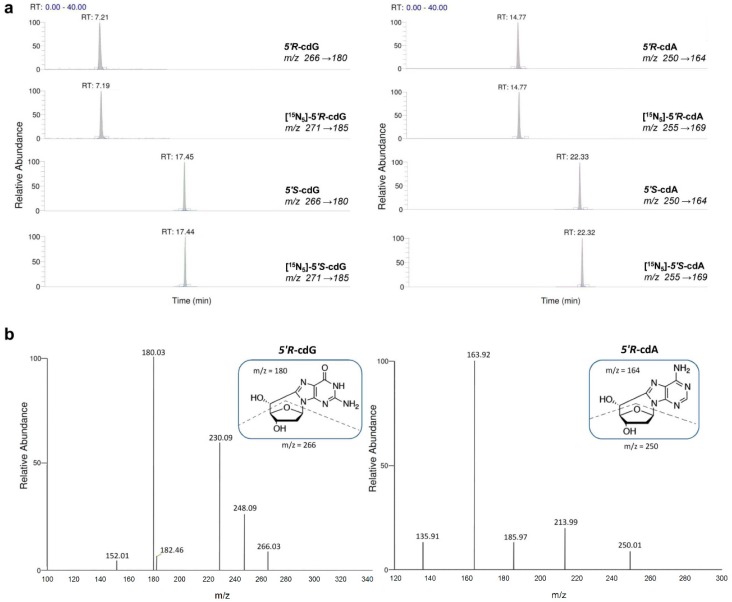
LC-MS/MS analysis. (**a**) MRM chromatograms of quantification ions obtained by LC–MS/MS analysis: Quantification transitions *m/z* 266→180 (5′*R*-cdG and 5′*S*-cdG), *m/z* 250→164 (5′*R*-cdA and 5′*S*-cdA), *m/z* 271→185 ([^15^*N*_5_]-5′*R*-cdG and [^15^*N*_5_]-5′*S*-cdG) and *m/z* 255→169 ([^15^*N*_5_]-5′*R*-cdA and [^15^*N*_5_]-5′*S*-cdA) (Table S1). (**b**) MS/MS fragmentation spectra (ESI-MS/MS of the [M+H]^+^ ion) of 5′*R*-cdG (*m/z* 266→180) and 5′*R*-cdA (*m/z* 250→164) lesions (similar fragment ions were observed for 5′*S*-cdG and 5′*S*-cdA lesions).

The quantification of the lesions was executed in two independent steps. Firstly, the sample was analysed via HPLC-UV system coupled with a fraction collector. According to this first clean-up step, the quantification of the unmodified nucleosides took place, based on their absorbance at 260 nm whereas, at their time-windows when the damaged lesions are eluted, fractions were collected and pooled. The concentrated samples containing the modified DNA damage lesions were injected subsequently to LC-MS/MS to be analyzed and quantified independently [21,22,25]. In the absence of the unmodified nucleosides, that arise solubility problems, the sample can be concentrated prior to LC-MS/MS analysis increasing the overall sensitivity of the quantification method. The use of isotopically labeled lesions maintains the reproducibility and the recovery of the quantification protocol within the levels that are generally accepted for reliability. Figure 2a highlights the MRM chromatograms of the corresponding mass transitions for 5′*R*-cdG, 5′*R*-cdA, 5′*S*-cdG and 5′*S*-cdA, as well as their isotope-labeled analogues [^15^*N*_5_]-5′*R*-cdG, [^15^*N*_5_]-5′*R*-cdA, [^15^*N*_5_]-5′*S*-cdG and [^15^*N*_5_]-5′*S*-cdA, which were acquired during LC-MS/MS analysis of the samples.

**Figure 3:**
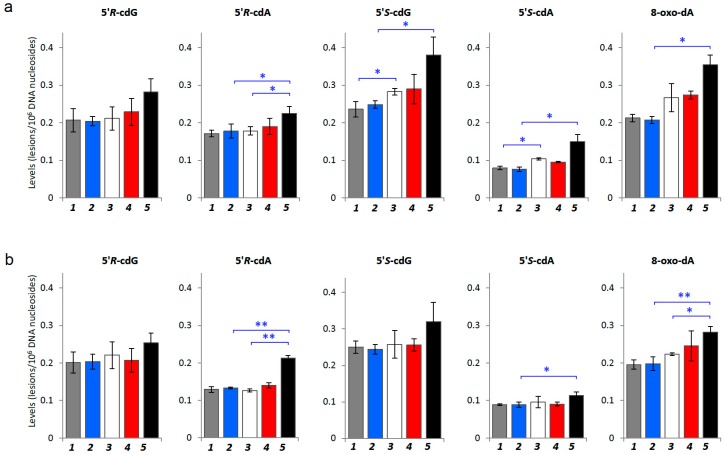
DNA lesion levels measurement by LC-MS/MS in tissues. (**a**) Levels of DNA lesions in genomic DNA isolated from liver of control SCID mice (*Group 1*: 4-weeks; *Group 2*: 17-weeks old) and tumor-bearing SCID mice (*Group 3*: 4-weeks old; *Group 4*: 5-weeks old; *Group 5*: 17-weeks old). (**b**) Levels of DNA lesions in genomic DNA isolated from kidney of control SCID mice (*Groups 1* and *2*) and tumor-bearing SCID mice (*Groups 3*, *4* and *5*). Statistical analysis was performed using *t*-test by comparing the means of control SCID and tumor-bearing SCID mice at each specific age point (Two-Sample Assuming Unequal Variances) and error bars represent standard deviation of the mean, calculated from three independent animals. * denotes a statistically significant difference (*p* < 0.05) and ** denotes a statistically significant difference (*p* < 0.005) between the animal groups.

Figure 2b illustrates the fragment ions of *m/z* 180 for cdG and 164 for cdA, which were obtained from the cleavage of both the *N*-glycosidic bond and C4′–C5′ bond of the 2-deoxyribose unit [7]. The 8-oxo-dA was eluted at 25.5 min and the *m/z* 268→152 transition for this lesion was monitored, accordingly. As a premise of this work, we estimated whether differences of such DNA damage lesions exist between control SCID mice and healthy Swiss mice, since no data are available in literature. As shown in Table S2 (see Supplementary Materials) in both liver and kidney tissues the levels of cPu and 8-oxo-dA were 1.1–1.6 fold higher in SCID than in healthy Swiss mice. Then we examined the four cPu and 8-oxo-dA in the liver of 17 weeks old tumor-bearing animals (oldest animals) finding increased levels compared with 4- and 5-weeks-old diseased mice (Figure 3a). In particular, 5′*S*-cdG was the predominant lesion with levels of (0.28–0.38) × 10^6^ nucleobases followed by 5′*R*-cdG with levels of (0.21–0.28) × 10^6^ nucleobases. In the same Figure it can be observed that 5′*R*-cdA has statistically significant higher levels at terminal stages of tumor development than at 4 weeks (4 weeks compared to 17 weeks; *p* = 0.037). In general, the tumor-bearing mice showed substantially increased levels of cPu adducts in liver compared to the control mice (1.07–1.53-fold) (see Table 1). Statistically significant differences for 5′*S*-ScdA were found at 4 weeks aged SCID mice compared with diseased mice of the same age (*p* = 0.025,) as well as at 17-weeks-old SCID mice compared with 17-weeks-old xenografts for 5′*R*-cdA, 5′*S*-cdG, 5′*S*-cdA (*p* = 0.036; *p* = 0.041; *p* = 0.025, respectively), as summarized in Table 1 and Table 2. Higher levels of 8-oxo-dA were also present in liver of 17 weeks aged tumor-bearing mice compared to control SCID mice at the same weeks of age (*p* = 0.012; Figure 3a).

In kidney, similarly to liver, an increased accumulation of endogenously induced DNA damage was observed in tumor-bearing mice compared to SCID animals (Figure 3b). 5′*S*-cdG was again identified as the prevalent among the four cPu lesions, in SCID and diseased animals with detected levels of (0.24–0.2) × 10^6^ nucleobases and (0.26–0.32) × 10^6^ nucleobases, respectively. Statistically significant differences were indicated between 17-weeks old SCID and 17-weeks aged tumor-bearing mice (*p* = 0.00003, 5′*R*-cdA; *p* = 0.025, 5′*S*-cdA) and for 5′*R*-cdA levels (*p* = 0.0003) between early and late lifespan of tumor-bearing mice (Table 1 and Table 2). Higher levels of 8-oxo-dA were also present in kidney of 17-weeks aged tumor-bearing mice. Statistically significant differences in kidney were found among 17-weeks old SCID and 17 weeks old xenografts (*p* = 0.003), as well as comparing tumor-bearing animals of 4 weeks of age with tumor-bearing mice at 17-weeks of age (*p* = 0.022).

In summary, in SCID mice without tumors all the four cPu adducts levels were comparable, and differences were not statistically significant. Comparing the SCID (control) and diseased (xenografts) conditions, in diseased mice the levels of the four cyclopurines were 1.1-fold increased at 4-weeks old animals and at least 1.4-fold increased at 17-weeks-old diseased mice, both in liver and kidney tissues.

### 2.3. Fatty Acid-Based Membrane Lipidomics of Erythrocytes

Since the erythrocyte membrane composition in SCID mice is not known, we performed an initial comparison between control SCID mice and healthy Swiss mice (see Tables S3 and S4 in Supplementary Materials). As mentioned before, it is not the scope of this paper to evaluate the differences between healthy Swiss and control SCID mice, however an initial evaluation of the distribution of the RBC membrane fatty acid families in these two animal models was carried out, before examining the membrane fatty acid distribution during the tumor progression in the immuno-deficient model of SCID mice. We found only a few significant fatty acid changes during ageing in animals (i.e., palmitic acid 16:0 diminution in SCID mice, *p* = 0.0377, see Table S3) without extensive changes of the fatty acid families and indices, therefore we can exclude the ageing process as main factor for lipidome changes detected in our model. The membrane lipidomic analyses were performed in tumor-bearing mice at early stage of tumorigenesis (4- and 5-weeks-old mice) and at a final stage of tumor progression (tumor-bearing 17-weeks-old mice), as shown in Table 3.

**Figure 4:**
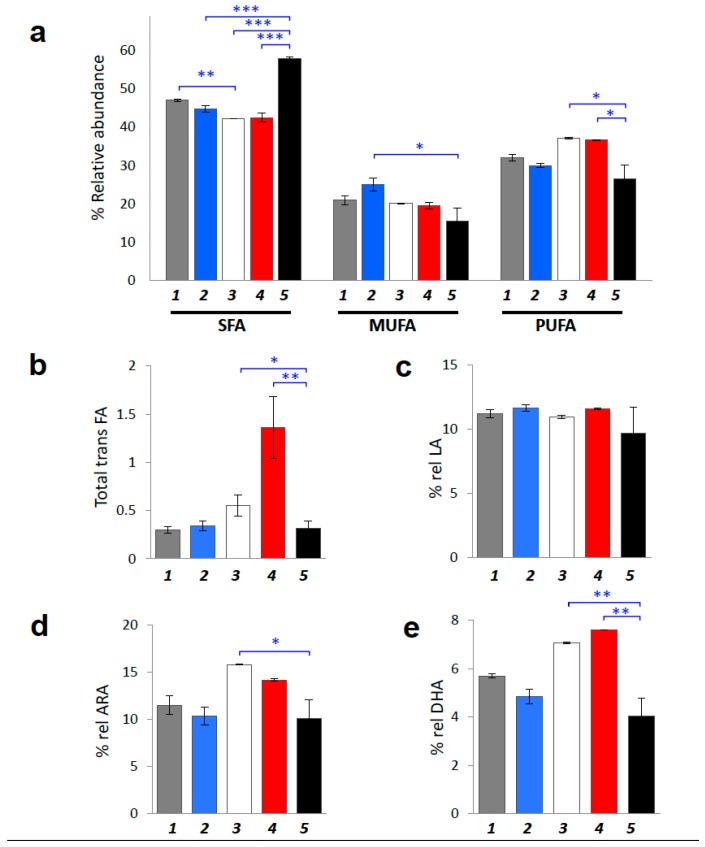
Fatty-acid based membrane lipidomic analysis. (**a**) Fatty acid composition grouped into families SFA, MUFA and PUFA of erythrocyte membrane at early and late stages of control SCID and tumor-bearing mice; comparison among control (4-weeks and 17-weeks old; *Group 1* and *Group 2*, respectively) and diseased mice (4-weeks old, 5-weeks old and 17-weeks old; *Group 3*, *Group 4* and *Group 5*, respectively). (**b**) Total *trans* fatty acids isomers of erythrocyte membrane among control SCID (*Group 1* and *Group 2*) and tumor-bearing mice (*Group 3, Group 4* and *Group 5*). (**c**) LA (18:2 ω-6) levels for the five distinct mice groups. (**d**) ARA (20:4 ω-6) levels for the five distinct mice groups. (**e**) DHA (22:6 ω-3) levels for the five distinct mice groups. Values represent mean ± SD (*n* = 3). * (*p* < 0.05), ** (*p* < 0.01), *** (*p* < 0.001).

In Table 4, fatty acid values are reported for the three families of SFA, MUFA and PUFA (graphically represented in Figure 4a), *trans* fatty acids (TFA) (graphically represented in Figure 4b), as well as for PUFA ω-3, PUFA ω-6 and their ratio. In Table 4 the calculated indices of unsaturation (UI) and peroxidation (PI) are also reported [54,55].

The following interesting differences emerged from the comparison between control and tumor-bearing SCID mice at different time points:

(A) Membrane lipidome profiles showed PUFA level decrease during tumor propagation. 17-weeks-old tumor-bearing mice are characterized by significant less PUFA content compared to the young (4- and 5-weeks-old) tumor-bearing age mice (*p* = 0.0071 and *p* = 0.0085, respectively). Tumor-bearing mice at early ages showed increased PUFA levels, becoming similar to healthy Swiss mice (compare Table 4 with Table S4). At 17-weeks of age tumor-bearing SCID mice showed the significant depletion of PUFA in RBC membrane phospholipids (*p* = 0.0019) with a corresponding increase of SFA (*p* < 0.0001). In particular, the relative percentage of SFA reached ca 58% over the total fatty acid composition in late stage tumor-bearing mice, the highest SFA percentage found compared to both control SCID and healthy Swiss mice (Table 4 and Table S4), and also compared to the percentage of SFA that corresponds to early stage tumor-bearing mice (ca. 42%; (*p* < 0.0001)).
(B) Some members of the ω-6 and ω-3 PUFA families (ARA and DHA graphically represented in Figure 4d,e, respectively) are also interesting membrane components to follow, showing significant depletion in tumor-bearing SCID mice after 17 weeks progress (*p* = 0.0333 and *p* = 0.0027, respectively). Remarkably, in all cases the PUFA changes did not involve the ω-6 precursor linoleic acid (LA: 9*cis*,12*cis*-C18:2), showing only a diminution trend at the late tumor stage (Table 3).
(C) Indexes of unsaturation and peroxidation are significantly decreased (UI and PI; *p* = 0.0014 and *p* = 0.0053, respectively). It is worth noting that neither in SCID mice nor in healthy Swiss mice the UI and PI indices were found significantly affected by the age (see Table 4 and Table S4). Moreover, in SCID mice of 4-weeks these indices were more comparable to the Swiss healthy than to the SCID mice (cfr., Table 4 with Table S4).
(D) The level of TFA in tumor-bearing SCID mice, was significantly increased at 5-weeks and again decreased in 17-weeks old tumor-bearing mice (*p* = 0.0074) (Table 4 and Figure 4c). No significant changes were revealed for control SCID mice while only in healthy Swiss mice the TFA were decreased during ageing (Table S4, *p* = 0.0006).

Finally, we estimated correlations between the changed level of the fatty acids and the ROS-induced DNA lesions, although we were aware that the number of animals (*n* = 3) used in this exploratory study would not be enough to observe statistical significance. The results are shown in Table S5 of Supplementary Materials. Indeed, we could not observe significance but it is worth noting the very high correlation scores (*r* > 90%) were observed for saturated fatty acids (16:0 and 18:0) as well as for arachidonic acid, EPA and DHA with the four DNA lesions (Table S5). In particular, the 5′*R* diastereoisomers of both cdA and cdG correlated only with arachidonic acid (*r* = −0.9444 and *r* = 0.9552, respectively), whereas the 5′*S* diastereoisomers correlated with SFA, EPA and DHA.

## 3. Discussion

**Figure 5:**
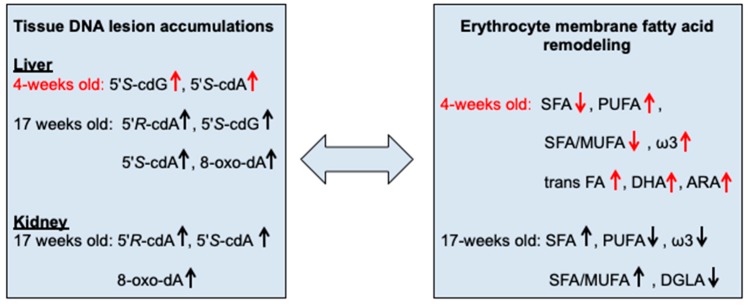
The diagram shows changes in the biological compartments found in the model of tumor-bearing SCID mice compared with SCID mice without tumor implantation at 4 weeks and 17 weeks of age; up-arrow: increase, down-arrow: decrease.

In the present study we envisaged the possibility to outline an integrated scenario of oxidatively-induced DNA damage and membrane remodeling occurring in a model of ageing and cancer progression. Ageing and cancer processes have often been related to each other, and the interplay of molecular changes, which affect the biology of ageing, can be informative in cancer biology [56]. We examined ROS-induced DNA and lipid transformations related to free radical reactivity, such as the four cPu lesions [7,8] and the membrane fatty acid remodeling, respectively [29,42,57]. The oxidatively-induced DNA lesions were determined in two distinct tissues (liver and kidney) in young and old tumor-bearing mice and paralleled with the membrane fatty acid profile obtained from the erythrocytes in the same animal groups. A glance at the level of the nucleic acid lesions and membrane fatty acids was obtained also for healthy Swiss mice compared to SCID mice, in order to evidence if changes are due to the ageing process (Tables S2, S3 and S4). Differences in ROS-induced DNA lesion levels between healthy Swiss mice and SCID mice could be attributed to the different (healthy and immuno-deficient) phenotypes of these animals. On this basis, it is reasonable to make comparison among treated and untreated SCID organisms. The schematic diagram in Figure 5 summarizes the results from this exploratory study.

Comparing DNA oxidative damage in tumor-bearing mice with those in control SCID mice, the cPu lesion profile is 1.1–1.4-fold increased at 4 and 17 weeks. In young tumor-bearing mice comparing 4 and 5 weeks, the overall tendency of the four DNA lesions is marginally elevated and not statistically significant, likely due to the short lifetime lapse of 7 days (see Figure 3). Tumor-bearing animals at seventeen weeks of age accumulated higher levels of cPu both in liver and kidney than younger tumor-bearing mice (Figure 3a,b). The quantities of cPu lesions were found to be slightly higher in liver than kidney, suggesting a higher levels of hydroxyl radicals in the liver organ. The tumor-dependent increases of the lesion levels in the liver and kidney of mice (Figure 3a,b) support the hypothesis that DNA repair mechanisms might be progressively impaired in these conditions.

In the same animals the membrane lipidomic profiles of erythrocytes were obtained in order to estimate signatures of fatty acid composition that can be indicative of the consequences of a high oxidation status which, together with DNA, can describe the membrane exposure through the PUFA consumption and the consequent membrane remodeling. This scenario is connected to health conditions such as tumor progression, since altered fatty acid metabolism and enhanced activity of fatty acid synthase and desaturase enzymes were previously reported [58]. In this study the erythrocyte membrane was used as the representative compartment for fatty acid availability in the body, including PUFA, which are important components of these membranes.

Considering the membrane fatty acid remodeling, late stage and early stage tumor-bearing mice were found significantly different with a decrease in PUFA (both ω-6 and ω-3 counterparts), Unsaturation Index (UI) and Peroxidation Index (PI) at late tumor progression. This decrease is accompanied by a significant increase of SFA. The lipidome profile in early stage tumor-bearing mice showed a PUFA content very similar to healthy Swiss mice, suggesting a role of PUFA in tumors for cancer risk [59] and its progression [60]. Moreover, an important distinction of two different levels of PUFA during tumor progression was highlighted: increased at early stages in tumor-bearing SCID mice and similar to healthy Swiss mice (Table 4 and Table S4), and then lowered as the tumor progresses. Tumor tissue is highly correlated with oxidative stress [28,29] therefore the PUFA diminution, probably connected with lipid peroxidation in cell membranes, is expected. However, it is worth underlining that in our samples the level of LA, the ω-6 precursor, is not significantly affected confirming that oxidative processes are localized to tissues rich of ARA and DHA. Indeed, these two PUFAs represent the most relevant lipids in neuronal cells and the mice were inoculated with human glioma cell line [61]. Our study was based on erythrocyte membranes as an easily withdrawn biological specimen to examine. Being both a “circulating” tissue resulting from a balance between the reactive oxygen species and antioxidant status [23], and a “reporter” on the status of other tissues, which are not easily reachable, the fatty-acid based membrane lipidomics of erythrocytes can be seen as a useful tool to combine with other relevant metabolic and cellular information in animal models. In this case, it is also worth recalling that erythrocyte and liver cells are reported to be similar both in the membrane fatty acid composition [61] and in the mean lifetime (120 and 180 days, respectively). Another process correlated with the free radical stress is the geometrical cis-trans lipid isomerization [29,42], as observed in the membranes of the animals of our study. The trans fatty acid levels detected in tumor-bearing mice changed significantly in the fifth week, probably related to an increased isomerizing radical production after this time, whereas at the beginning and late tumor stages a lower isomerizing effect is seen (see Table 4 and Figure 4b). As matter of facts, the role of trans fatty acids (TFA) in cancer is still controversial [62] and in this work we do not give any conclusive explanation to such behavior. ARA and DHA, which are the main long chain PUFA counterparts in membrane, are prone to oxidation and show significant decrease on late stage tumor-bearing mice. PUFA can also be connected with their role for lipid signaling. For instance, the arachidonic acid moieties are removed from membrane phospholipids by the enzyme PLA2, giving rise to signaling cascades that are known to lead to apoptosis evasion and tumor progression [57,63]. The consumption of unsaturated fatty acids is accompanied by increased peroxidation of long-chain unsaturated fatty acids which results in the production of reactive aldehydes, most of them having a strong pro-apoptotic properties [64]. It is interesting to note that during tumor development the PUFA decrease is accompanied by an increase of SFA, which indicates the availability of the latter fatty acids from the de novo fatty acid biosynthesis. Indeed, many studies reported an enhanced FASN activity in tumor progression, accompanied by an increase of desaturase activity, although this latter process is not observed in our animal model [33]. In a comprehensive vision, lipid membrane remodeling could act as a double-edged sword, on one hand accelerating tumor progression, and on the other hand, slowing-down tumor growth by stimulating apoptosis.

When correlations were estimated between fatty acid levels and ROS-induced DNA lesions (Table S5) the low number of animals (*n* = 3) did not allow to get statistical significance although correlation scores *r* were higher than 90% for saturated fatty acids (16:0 and 18:0) as well as for arachidonic acid, EPA and DHA with the DNA lesions. The 5′*R* diastereoisomers of cdA and cdG were predominant in kidney and liver tissues and their correlation with arachidonic acid could indicate a role of the inflammation process for the prevalence of such isomers. Further studies are needed to extend models and corroborate this hypothesis linking membrane and DNA damages.

## 4. Materials and Methods

### 4.1. Materials

Nuclease P1 from *Penicillium citrinum*, phosphodieasterase I and II, alkaline phosphatase from bovine intestinal mucosa, DNase I and DNase II, benzonase 99%, BHT, deferoxamine mesylate and pentostatin were purchased from Sigma-Aldrich (Steinheim, Germany). RNase T1 was from Thermo Fisher Scientific (Waltham, MA, USA) and RNase A from Roche Diagnostic GmbH, (Mannheim, Germany). The 3 kDa cut-off filters were obtained from Millipore (Bedford, OH, USA). Solvents (HPLC-grade) were purchased from Fisher Scientific. 2′-Deoxyadenosine monohydrate were purchased from Berry & Associates Inc. (Dexter, NY, USA). Isotopic labeled internal standards of 5′*R*-cdA, 5′*S*-cdA, 5′*R*-cdG, 5′*S*-cdG and 8-oxo-dA (see Supplementary Materials) were prepared according to the previously reported procedures [21]. Ultrapure water (18.3 MΩ.cm) distilled and deionized water (Milli-Q water) were purified by a Milli-Q system (Merck-Millipore, Bedford, OH, USA). Chloroform, methanol and *n*-hexane were purchased from Merck (HPLC-grade). Anhydrous sodium sulfate (Na_2_SO_4_) was purchased from Carlo Erba (Val de Reuil Cedex, France). All fatty acid methyl esters (FAME) used as reference standard for GC analyses were purchased from Sigma-Aldrich or Fluka (Steinheim, Germany) without further purification. Analytical silica gel thin-layer chromatography was performed on Merck silica gel 60 plates.

### 4.2. Animal Studies

Mice were housed at SOL-GEL laboratory at the NCSR “Demokritos”. Female SCID mice and normal healthy Swiss mice were housed in groups of three per cage under positive pressure in polysulfone type IIL individual ventilated cages (Sealsafe, Tecniplast, Buguggiate, Italy). Room temperature and relative humidity were 24 ± 2 °C and 55 ± 10% respectively. All animals in the facility were screened regularly according to the Federation of European Laboratory Animal Science Associations’ recommendations and were found free of the respective pathogens. Mice had ad libitum access to water and food. SCID mice were inoculated with U87MG human brain glioblastoma cells just above the right flank, as previously described [65,66]. Humane endpoints were predetermined (tumor volume over 1.2 cm, severe compromise of the welfare of the animals, and body weight loss over 20%). The tumor volume and mice weight were monitored once a week with an automatic caliper and scale. The tumor volume, body weight and the survival rate were calculated in different time intervals (Tables S6 and S7). Ethical statement: All protocols were approved by the General Directorate of Veterinary Services Athens, according to Greek legislation (Presidential Degree 160/1991) in compliance with the European Community Directive 609/1986, and Law 2015/1992 for the protection of vertebrate animals used for experimental or other scientific purposes, 123/1986.

### 4.3. Cell Lines, Cell Culture Conditions and Xenograft Construction

High glucose Dulbecco’s modified Eagle Medium (DMEM) was purchased from Sigma (Steinheim, Germany). Trypsin-EDTA, L-glutamine, penicillin–streptomycin solution and heat inactivated fetal bovine serum (FBS) were obtained from Biochrom KG (Berlin, Germany). U87MG brain glioblastoma was obtained from the American Type Culture Collection (ATCC; Manassas, VA, USA) and cultured as monolayers at 37 °C in a humidified atmosphere of 5% (*v*/*v*) CO_2_ and 95% relative humidity. Cells were seeded in 75-cm^2^ plastic tissue culture flasks and cultured in DMEM supplemented with 10% FBS, washed with phosphate buffered saline (PBS) and were harvested by trypsinization with 0.05% (*w*/*v*) trypsin in PBS containing 0.02% (*w*/*v*) EDTA. SCID mice were xenografted at two weeks of age with U87MG cells subcutaneously at the right side of the thorax. Tumors were inoculated after injection of 6 × 10^6^ U87MG cells in SCID mice, which were previously grown in DMEM.

### 4.4. Genomic DNA Isolation

Mice were sacrificed under deep ether anesthesia and the tissues were promptly removed, placed in a polypropylene tube, immediately flash-frozen in liquid nitrogen and stored at −80 °C. Genomic DNA from frozen tissues was isolated using a high-salt extraction method. The frozen samples were suspended in 1 mL of tissue lysis buffer containing 10 mM Tris pH 7.5, 2 mM EDTA, 400 mM NaCl, 1% SDS (*w*/*v*), 200 μg/mL Proteinase K, 100 μΜ deferoxamine, 100 μΜ butylated hydroxytoluene and incubated at 55 °C overnight. Half volume of saturated NaCl solution was added to the digestion mixture, incubated at 55 °C for 15 min and then centrifuged at 10,000 rpm for 30 min at room temperature. The nucleic acids in the supernatant were precipitated from absolute ethanol (molecular biology grade, Merck-Millipore). RNase A (10 mg/mL) and RNase T1 (25 units/μL) were added to the nucleic acid mixture and after 1h incubation at 37 °C, an extraction step with an equal volume of chloroform/isoamyl alcohol (24:1, *v*/*v*) and ethanol precipitation step were performed. After centrifugation at 10,000 rpm for 10 min, the resulting DNA pellet was washed twice with 70% cold ethanol, allowed to dry in a fume hood to evaporate all of the ethanol and resuspended in deionized water.

### 4.5. Enzymatic Digestion to Nucleosides

50 μg isolated DNA were dissolved in 100 μL of Ar flushed 10 mM Tris-HCl (pH 7.9), containing 10 mM MgCl_2_, 50 mM NaCl, 0.2 mM pentostatin, 5 μM BHT and 3 mM deferoxamine and the internal standards were added ([^15^*N*_5_]-5′*S*-cdA, [^15^*N*_5_]-5′*R*-cdA, [^15^*N*_5_]-5′*S*-cdG, [^15^*N*_5_]-5′R-cdG and [^15^*N*_5_]-8-oxo-dA) as previously described [21,22,67,68] (see Figure S1). 3 U of benzonase (in 20 mM Tris-HCl pH 8.0, 2 mM MgCl_2_ and 20 mM NaCl), 4 mU phosphodiesterase I, 3 U DNAse I, 2 mU of phosphodiesterase II and 2 U of alkaline phosphatase were added and the mixture was incubated at 37 °C. After 21 h, 35 μL of Ar flushed buffer containing 0.3 M AcONa (pH 5.6) and 10 mM ZnCl_2_ were added along with 0.5 U of Nuclease P1 (in 30 mM AcONa pH 5.3, 5 mM ZnCl_2_ and 50 mM NaCl), 4 mU PDE II and 125 mU of DNAse II and the mixture was further incubated at 37 °C for extra 21 h. A step-quenching with 1% formic acid solution (final pH~7) was followed, the digestion mixture was placed in a microspin filter (3 kDa) and the enzymes were filtered off by centrifugation at 14,000× *g* (4 °C) for 20 min. Subsequently, the filtrate was freeze-dried before HPLC analysis, clean-up and enrichment.

### 4.6. Analysis by High Performance Liquid Chromatography, Clean-Up and Enrichment

HPLC-UV clean-up and enrichment of the enzyme free samples were performed on a 4.6 mm × 150 mm Atlantis^®^ dC18 100 Å column (5 μm particle size, Waters, Milford, MA, USA), loaded with a 4.6 mm × 20 mm Guard Column 2 pK (Atlantis^®^ dC18 5 μm) on a Waters Alliance^®^ HPLC System (Waters e2695Separations Module, including a Waters 2998 Photodiode Array (PDA) detector as previously described [22]. The gradient program used an eluent composed by 2 mM ammonium formate, acetonitrile and methanol, while the fractions containing the lesions were collected, freeze-dried, pooled, freeze-dried again, and redissolved in Milli-Q water before been injected for LC-MS/MS analysis [21,67,68].

### 4.7. Measurement of Modified Nucleosides by LC-ESI-MS/MS

An LC-MS/MS system Finnigan TSQ Quantum Discovery Max triple-stage quadrupole mass spectrometer (Thermo, Waltham, MA, USA), equipped with electrospray ionization (ESI) source in positive mode was employed for the detection and quantification of the lesions in the enzymatically digested DNA samples. Separation of target analytes was achieved with a Finnigan Surveyor LC system, equipped with a Finnigan Surveyor AS autosampler (Thermo). The Xcalibur software 2.1 SP 1160 was used to control the mass spectrometric parameters and for data acquisition. The collected fractions after HPLC analysis were subsequently injected to the LC-MS/MS system loaded with a 2.1 mm × 150 mm Atlantis^®^ dC18, 100 Å column (3 μm particle size, Waters) guarded by a 2.1 mm × 10 mm Guard Column 2pK (Atlantis^®^ dC18 3 μm, Waters). The gradient elution program used for the chromatographic separation of the DNA lesions initiated with 99% of 2 mM ammonium formate (solvent A) and 1% acetonitrile (solvent B) (held for 1 min), increasing solvent B from 1% to 9.8% within 20 min and then immediately to 15% solvent B (held for 5 min), closing with initial conditions for 10 min re-equilibration. The flow rate remained constant at 0.2 mL/min, the injection volume was 50 μL and column temperature was set at 30 °C. Detection was performed in multiple reaction monitoring mode (MRM) using the two most intense and characteristic precursor/product ion transitions for each DNA lesion [21].

### 4.8. RBC Fatty Acid-Based Membrane Lipidomic Analysis

Blood was withdrawn from deeply ether-anesthetized animals and collected in K_2_EDTA treated tubes. 200 μL whole blood from mice were centrifuged at 4000 rpm for 5 min at 4 °C to remove plasma fraction. The red blood cell (RBC) pellet was then resuspended in pure water and centrifuged at 14,000 rpm for 15 min at 4 °C. Membrane pellet was dissolved in 2:1 chloroform: methanol and examined by thin layer chromatography (*n*-hexane/diethyl ether/acetic acid 70/30/1) to determine the purity of the phospholipid fraction. The phospholipid extract was then treated with 0.5 M KOH/MeOH for 10 min at room temperature under stirring for the derivatization of fatty acid residues of the phospholipids into their corresponding fatty acid methyl esters (FAME). After this transesterification step, FAME were extracted with *n*-hexane; *n*-hexane phase was dehydrated with anhydrous Na_2_SO_4_, evaporated and analyzed by gas chromatography (Agilent 6850, Milan, Italy) equipped with a 60 m × 0.25 mm × 0.25 μm (50%-cyanopropyl)-methylpolysiloxane column (DB23, Agilent, Santa Clara, CA, USA), a flame ionization detector (FID), with injector temperature at 230 °C and split injection 50:1. Oven temperature started from 165 °C, held for 3 min, followed by an increase of 1 °C/min up to 195 °C, held for 40 min, followed by a second increase of 10 °C/min up to 240 °C, and held for 10 min. A constant pressure mode (29 psi) with helium as carrier gas was used. Methyl esters were identified by comparison with the retention times of commercially available standards or trans fatty acid references, obtained as described elsewhere) as described previously [69]. The list of the examined FAME (corresponding to chromatographic peak areas >97%) in RBC membrane phospholipid are reported in Table 3 as % relative percentages ± SD (standard deviation).

### 4.9. Statistical Analysis

All measurements were performed in triplicate and the data were expressed as mean ± standard deviation (SD). The unpaired *t*-test was used for statistical analysis and a two-tailed *p*-value < 0.05 and *p*-value < 0.005 were considered to indicate a statistical significant difference. One-way ANOVA test was also performed. All the values resulting from the quantitative analysis of the lesions are summarized in Table 1, showing the mean value (± standard deviation) of the lesions’ levels based on the accurate measurement of three DNA samples isolated from three independent tissues. The results of fatty acids are presented in Table 3 and Table 4 as mean ± SD from at least three replicates. Correlations between DNA lesions and membrane fatty acid levels were evaluated by parametric two-tailed Pearson test and are reported in Table S5.

## 5. Conclusions

In this work we provide an integrated scenario of molecular changes in DNA nucleobases and fatty acids of RBC membrane lipids accompanying tumor progression. In 17-weeks-old tumor-bearing mice the oxidatively-induced DNA damage lesions are increased compared to 4/5-weeks old tumor-bearing animals, with statistical significance of 5′*R*-cdA in liver and 5′*R*-cdA and 8-oxo-dA in kidney. This is paralleled with a membrane fatty acid profile pointing out the role of PUFA, with levels of ω-6 and ω-3 fatty acids, in particular ARA and DHA, and their decrease between early and late stage of tumor occurrence. In conclusion, for the first time the levels of some oxidatively-induced DNA lesions and erythrocyte membrane fatty acid composition have been determined simultaneously in a living model, evidencing the tumor contribution to the progressive molecular insult. The integrated approach can contribute to better define the metabolic scenario combining membrane lipid remodeling and oxidatively-induced DNA adducts in disorders with elevated radical reactivity and stress status, such as carcinogenesis, inflammation and ageing.

## Figures and Tables

**Table 1:** The levels (lesions/10^6^ nucleosides) of 5′*S*-cdA, 5′*R*-cdA, 5′*S*-cdG, 5′*R*-cdG and 8-oxo-dA in genomic DNA of liver and kidney of control SCID mice and tumor-bearing SCID mice in each age point. The numbers in the boxes represent the mean value (± standard deviation) of cPu and 8-oxo-dA levels from the measurement of three DNA samples isolated from three independent animals of each tissue. w: week.

Mice (Age, Week)	5′R-cdG	5′R-cdA	5′S-cdG	5′S-cdA	8-Oxo-dA
Liver					
Control (4 w)	0.207 ± 0.031	0.171 ± 0.009	0.236 ± 0.021	0.079 ± 0.005	0.212 ± 0.010
Control (17 w)	0.204 ± 0.012	0.178 ± 0.019	0.248 ± 0.010	0.076 ± 0.006	0.207 ± 0.009
Tumor-bearing (4 w)	0.211 ± 0.031	0.178 ± 0.011	0.283 ± 0.009	0.103 ± 0.003	0.267 ± 0.037
Tumor-bearing (5 w)	0.229 ± 0.035	0.190 ± 0.021	0.290 ± 0.039	0.095 ± 0.002	0.274 ± 0.011
Tumor-bearing (17 w)	0.282 ± 0.035	0.225 ± 0.018	0.381 ± 0.047	0.149 ± 0.020	0.354 ± 0.027
Kidney					
Control (4 w)	0.201 ± 0.028	0.129 ± 0.008	0.250 ± 0.017	0.089 ± 0.002	0.196 ± 0.012
Control (17 w)	0.204 ± 0.020	0.133 ± 0.003	0.244 ± 0.013	0.089 ± 0.007	0.198 ± 0.018
Tumor-bearing (4 w)	0.221 ± 0.036	0.126 ± 0.004	0.258 ± 0.038	0.095 ± 0.015	0.223 ± 0.003
Tumor-bearing (5 w)	0.207 ± 0.031	0.140 ± 0.007	0.256 ± 0.017	0.090 ± 0.006	0.246 ± 0.040
Tumor-bearing (17 w)	0.254 ± 0.027	0.213 ± 0.006	0.320 ± 0.053	0.113 ± 0.010	0.283 ± 0.015

**Table 2:** Values of *t*-test (Two-Sample Assuming Unequal Variances) are given by comparing the means of lesions in each specific age point of mice in liver and kidney, respectively. In the same table, *p* value is included, which represent the comparison among all the groups after conducting one-way ANOVA test in parallel with multiple comparisons of all groups (*n* = 3), w: week.

Mice (Age, Week)	5′R-cdG	5′R-cdA	5′S-cdG	5′S-cdA	8-Oxo-dA
Liver					
Control (4 w) vs. Tumor-bearing (4 w)	0.880	0.517	0.040	0.006	0.293
Control (17 w) vs. Tumor-bearing (17 w)	0.067	0.036	0.041	0.025	0.012
Tumor-bearing (4 w) vs. Tumor-bearing (5 w)	0.599	0.482	0.793	0.060	0.841
Tumor-bearing (4 w) vs. Tumor-bearing (17 w)	0.141	0.037	0.072	0.057	0.104
*p* value (ANOVA)	0.0568	0.0237	0.0022	<0.0001	<0.0001
Kidney					
Control (4 w) vs. Tumor-bearing (4 w)	0.571	0.663	0.836	0.651	0.065
Control (17 w) vs. Tumor-bearing (17 w)	0.059	0.000	0.136	0.025	0.003
Tumor-bearing (4 w) vs. Tumor-bearing (5 w)	0.699	0.073	0.955	0.734	0.438
Tumor-bearing (4 w) vs. Tumor-bearing (17 w)	0.383	0.000	0.222	0.289	0.022
*p* value (ANOVA)	0.2107	<0.0001	0.0768	0.0244	0.0055

**Table 3:** Relative percentages (% rel) of fatty acid methyl esters (FAME) from red blood cell (RBC) membrane of control SCID mice at different age points (4 weeks and 17 weeks) and tumor-bearing SCID mice at different age points (4 weeks, 5 weeks and 17 weeks).

FAME	Control (4 w)	Control (17 w)	Tumor-Bearing (4 w)	Tumor-Bearing (5 w)	Tumor-Bearing (17 w)	*p* Value
14:0	0.57 ± 0.06	0.55 ± 0.14	0.31 ± 0.02	0.33 ± 0.02	0.54 ± 0.05	0.0103
15:0	0.15 ± 0.04	0.15 ± 0.02	0.36 ± 0.05	0.46 ± 0.06	0.09 ± 0.07	0.0142
16:0	35.38 ± 0.46	31.78 ± 0.55	28.61 ± 0.07	29.23 ± 0.91	38.52 ± 1.90	0.0053
16:1c6	0.23 ± 0.02	0.23 ± 0.05	0.26 ± 0.01	0.37 ± 0.08	0.22 ± 0.07	0.2394
16:1c9	1.66 ± 0.36	2.89 ± 0.38	1.47 ± 0.21	1.37 ± 0.05	1.15 ± 0.65	0.8269
17:0	0.17 ± 0.00	0.16 ± 0.02	0.33 ± 0.01	0.45 ± 0.01	0.32 ± 0.04	0.0357
18:0	10.73 ± 0.09	12.11 ± 0.47	12.56 ± 0.06	12.08 ± 0.23	18.39 ± 2.02	0.0267
trans 18:1	nd	nd	0.20 ± 0.03	0.24 ± 0.07	0.03 ± 0.04	0.0379
18:1c9	15.87 ± 0.95	19.09 ± 1.20	16.05 ± 0.06	15.41 ± 0.53	12.10 ± 2.75	0.2525
18:1c11	2.70 ± 0.06	2.46 ± 0.07	1.94 ± 0.08	1.99 ± 0.12	1.81 ± 0.11	0.4285
trans 18:2	0.10 ± 0.00	0.10 ± 0.01	0.28 ± 0.01	0.44 ± 0.09	0.14 ± 0.05	0.0437
18:2 ω-6	11.22 ± 0.30	11.67 ± 0.25	10.97 ± 0.12	11.61 ± 0.07	9.67 ± 2.05	0.5189
18:3 ω-6	0.05 ± 0.05	0.16 ± 0.02	0.18 ± 0.01	0.09 ± 0.09	0.02 ± 0.03	0.1602
18:3 ω-3	0.18 ± 0.04	0.18 ± 0.00	0.19 ± 0.03	0.26 ± 0.07	0.17 ± 0.04	0.3714
20:1c11	0.48 ± 0.02	0.39 ± 0.04	0.37 ± 0.01	0.40 ± 0.01	0.24 ± 0.03	0.0089
20:2 ω-6	0.55 ± 0.02	0.39 ± 0.07	0.35 ± 0.03	0.33 ± 0.02	0.35 ± 0.15	0.9857
20:3 ω-6	1.22 ± 0.01	1.14 ± 0.13	1.26 ± 0.10	1.26 ± 0.01	0.80 ± 0.08	0.0088
trans 20:4	0.20 ± 0.03	0.24 ± 0.04	0.07 ± 0.07	0.68 ± 0.15	0.15 ± 0.05	0.0134
20:4 ω-6	11.53 ± 0.99	10.40 ± 0.95	15.81 ± 0.04	14.20 ± 0.09	10.07 ± 2.01	0.0463
20:5 ω-3	0.43 ± 0.03	0.42 ± 0.03	0.52 ± 0.03	0.53 ± 0.04	0.32 ± 0.07	0.0443
22:5 ω-3	0.89 ± 0.05	0.67 ± 0.04	0.76 ± 0.06	0.72 ± 0.05	0.87 ± 0.18	0.5865
22:6 ω-3	5.70 ± 0.08	4.84 ± 0.30	7.07 ± 0.03	7.60 ± 0.01	4.04 ± 0.74	0.0064

*p* value represents the comparison after conducting one-way ANOVA test in parallel with multiple comparisons of all groups (*n* = 3). nd: not detected.

**Table 4:** Fatty acid families and indices (obtained from the fatty acid values of Table 3 of control SCID mice (4- and 17-weeks) and tumor-bearing SCID mice at different age points (4-, 5- and 17-weeks).

Fatty Acid Families and Indices	Control (4 w)	Control (17 w)	Tumor-Bearing (4 w)	Tumor-Bearing (5 w)	Tumor-Bearing (17 w)	*p* Value
SFA	46.99 ± 0.35	44.75 ± 0.93	42.16 ± 0.03	42.54 ± 1.07	57.86 ± 0.44	<0.0001
MUFA	20.94 ± 1.20	25.06 ± 1.60	20.08 ± 0.08	19.53 ± 0.77	15.54 ± 3.34	0.0658
PUFA	32.07 ± 0.85	30.00 ± 0.48	37.09 ± 0.13	36.58 ± 0.01	26.60 ± 3.58	0.0190
PUFA ω-6	24.86 ± 0.65	23.89 ± 0.77	28.56 ± 0.04	27.48 ± 0.04	21.21 ± 3.47	0.0960
PUFA ω-3	7.21 ± 0.20	6.11 ± 0.29	8.54 ± 0.09	9.11 ± 0.03	5.39 ± 0.94	0.0054
ω-6/ω-3	3.45 ± 0.01	3.93 ± 0.31	3.35 ± 0.03	3.02 ± 0.01	4.05 ± 1.02	0.5875
SFA/MUFA	2.25 ± 0.15	1.80 ± 0.15	2.10 ± 0.01	2.18 ± 0.14	3.89 ± 0.76	0.0281
total trans	0.30 ± 0.03	0.34 ± 0.05	0.55 ± 0.11	1.36 ± 0.32	0.32 ± 0.07	0.0074
UI	135.06 ± 3.01	128.63 ± 0.47	159.61 ± 0.53	156.82 ± 0.63	109.00 ± 10.19	0.0014
PI	114.39 ± 4.76	101.76 ± 1.50	142.52 ± 0.60	140.68 ± 0.24	92.10 ± 13.59	0.0053

*p* value represents the comparison among all the groups after conducting one-way ANOVA test in parallel with multiple comparisons of all groups (*n* = 3).

## Supplementary Materials

The following are available online at https://www.mdpi.com/2072-6694/11/4/480/s1, Figure S1: Structures of DNA lesions and ^15^*N* isotopically labeled internal standards, Figure S2: PUFA moieties including arachidonic acid and its mono-*trans* isomers, linoleic acid and docosahexaenoic acid, Table S1: MRM transitions of the lesions, Table S2: Lesion levels in tissues, Table S3: Relative percentages (% rel) of fatty acid methyl esters (FAME) from red blood cell (RBC) membrane of normal healthy (Swiss) mice and control SCID mice at different age points (4 weeks and 17 weeks), Table S4: Fatty acid families and indices of normal healthy (Swiss) mice at different age points (4 weeks and 17 weeks), Table S5: Correlation between significantly altered fatty acids and DNA lesions, Table S6: Tumor growth progress through time of tumor-bearing SCID mice, Table S7: Weight of control SCID mice and tumor-bearing SCID mice.

## Abbreviations

16:0	palmitic acid
18:0	stearic acid
ARA	arachidonic acid
BER	base excision repair
cdA	5′ 8-cyclo-2′-deoxyadenosine
cdG	5′ 8-cyclo-2′-deoxyguanosine
cPu	purine 5′ 8-cyclo-2′-deoxynucleoside
DHA	docosahexaenoic acid
DGLA	dihomo-gamma-linolenic acid
EPA	eicosapentaenoic acid
FAME	fatty acid methyl esters
FASN	fatty acid synthase
GC	gas chromatography
LA	linoleic acid
LC-MS/MS	liquid chromatography tandem mass spectrometry
MUFA	monounsaturated fatty acids
NER	nucleotide excision repair
8-oxo-dA	8-oxo-7,8-dihydro-2′-deoxyadenosine
PI	peroxidation index
PUFA	polyunsaturated fatty acids
RBC	red blood cells
ROS	reactive oxygen species
SCID	severe combined immunodeficient
SFA	saturated fatty acids
TFA	trans fatty acids
UI	unsaturation index
