# Supplementary material for: Membrane Lipidome Reorganization and Accumulation of Tissue DNA Lesions in Tumor-Bearing Mice: An Exploratory Study
Source: Cancers (Basel). 2019 Apr 4;11(4):480. doi: 10.3390/cancers11040480 (PMC6520748; doi:10.3390/cancers11040480)
Supplement: Supplementary file 1 [file cancers-11-00480-s001.pdf]

Supplementary Materials

Membrane lipidome reorganization and accumulation of tissue DNA lesions in tumor-bearing mice: An exploratory study

Marios G. Krokidis, Maria Louka, Eleni K. Efthimiadou, Sevasti-Kiriaki Zervou, Kyriakos Papadopoulos, Anastasia Hiskia, Carla Ferreri and Chryssostomos Chatgilialoglu*

*CONTACT: chrys@isof.cnr.it

List of Content:

1. Structures of DNA lesions and ^{15}N isotopically labeled internal standards.
2. PUFA moieties including arachidonic acid and its mono-*trans* isomers, linoleic acid and docosahexaenoic acid
3. MRM transitions of the lesions.
4. Lesion levels in tissues.
5. Fatty acid methyl ester levels in RBC membrane.
6. Correlation between significantly altered fatty acids and DNA lesions
7. Tumor size and weight of mice

1. **Structures of DNA lesions and ^{15}N isotopically labeled internal standards.**

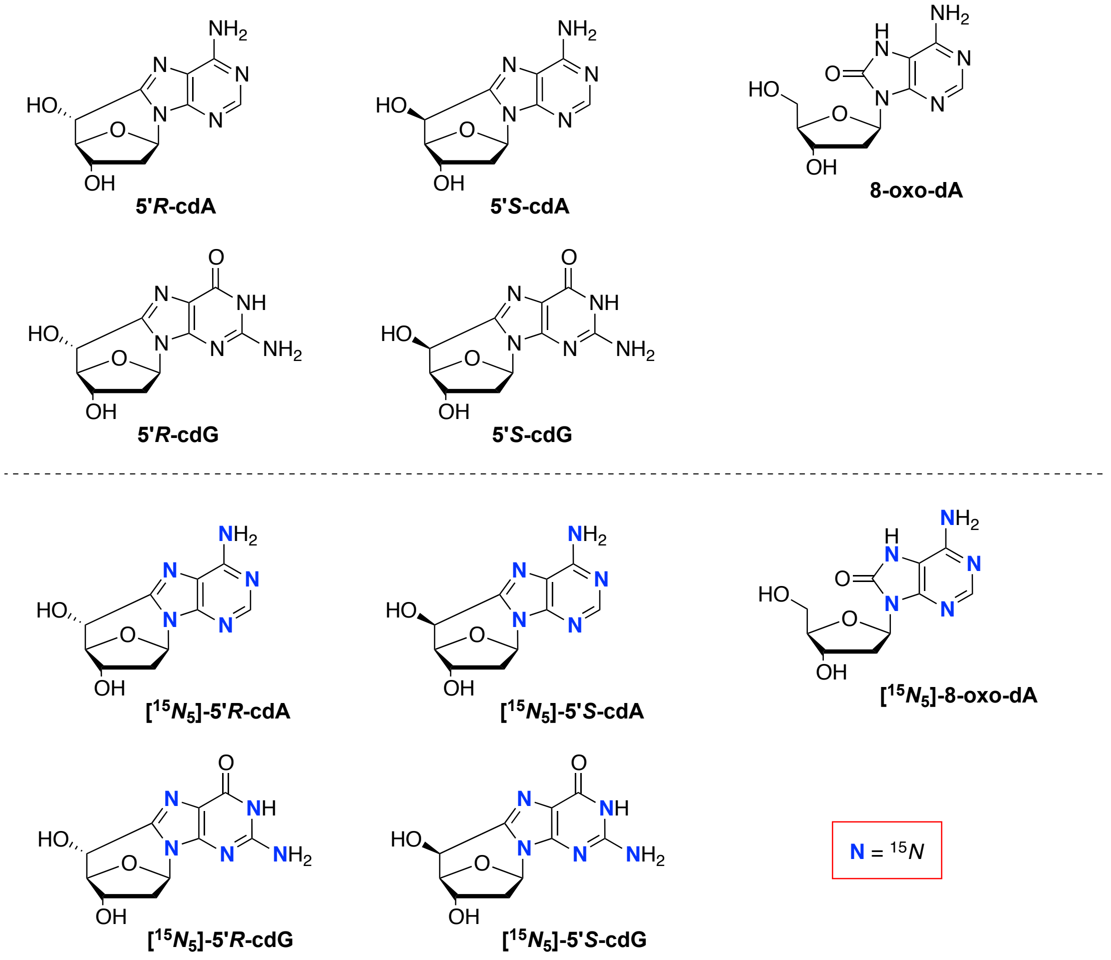

Figure S1. (Upper) Structures of 5′,8-cyclo-2′-deoxyadenosine (cdA) and 5′,8-cyclo-2′-deoxyguanosine (cdG) in their 5'*R* and 5'*S* diastereomeric forms, and 8-oxo-2′-deoxyadenosine (8-oxo-dA). (Lower) ^{15}N isotopically labeled compounds.

2. **PUFA moieties including arachidonic acid and its mono-*trans* isomers, linoleic acid and docosahexaenoic acid.**

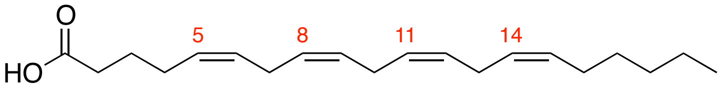

arachidonic acid (ARA) or 5*cis*,8*cis*,11*cis*,14*cis*–C20:4

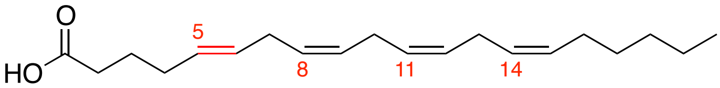

5*trans*,8*cis*,11*cis*,14*cis*–C20:4

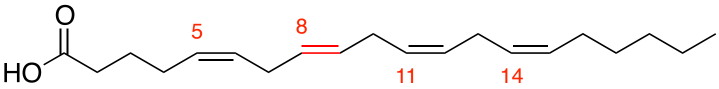

5*cis*,8*trans*,11*cis*,14*cis*–C20:4

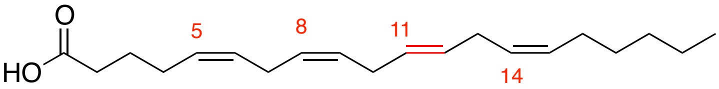

5*cis*,8*cis*,11*trans*,14*cis*–C20:4

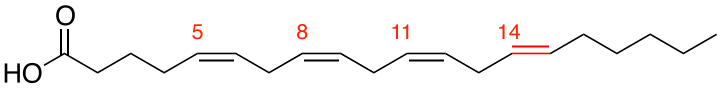

5*cis*,8*cis*,11*cis*,14*trans*–C20:4

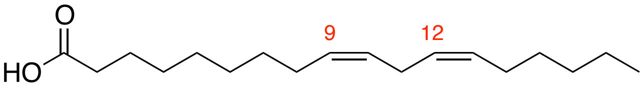

linoleic acid (LA)

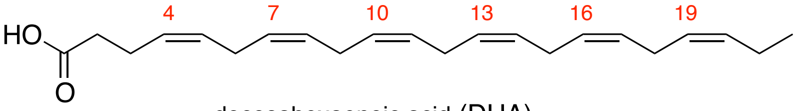

docosahexaenoic acid (DHA)

Figure S2. (Upper) Arachidonic acid (ARA) and its four mono-*trans* isomers. (Lower) The structures of LA and DHA.

3. MRM transitions of the lesions

Table S1. A list of MRM transitions employed for the quantifications of the oxidatively induced DNA lesions and their corresponding stable isotope labeled analogues.

	Precursor ion m/z	Product ion m/z	Collision energy (V)
5'*R*-cdA	250	164	14
[$^{15}N_5$]-5'*R*-cdA	255	169	14
5'*S*-cdA	250	164	16
[$^{15}N_5$]- 5'*S*-cdA	255	169	16
5'*R*-cdG	266	180	18
[$^{15}N_5$]- 5'*R*-cdG	271	185	18
5'*S*-cdG	266	180	16
[$^{15}N_5$]- 5'*S*-cdG	271	185	16
8-oxo-dA	267	151	19
[$^{15}N_5$]- 8-oxo-dA	272	156	19

4. Lesion levels in tissues

Table S2. The levels (lesions/10^6 nucleosides) of 5'*S*-cdA, 5'*R*-cdA, 5'*S*-cdG, 5'*R*-cdG and 8-oxo-dA in genomic DNA of liver and kidney of normal healthy (Swiss) and SCID mice (control) in each age point. The numbers in the boxes represent the mean value (± standard deviation) of cdPu and 8-oxo-dA levels of each tissues from the measurement of three DNA samples isolated from three independently animals.

Mice/age	5'R-cdG	5'R-cdA	5'S-cdG	5'S-cdA	8-oxo-dA
**liver**					
normal healthy 4w	0.162±0.020	0.121±0.031	0.190±0.041	0.067±0.001	0.163±0.018
normal healthy 17w	0.160±0.031	0.122±0.014	0.203±0.037	0.069±0.008	0.183±0.004
SCID 4w	0.207±0.031	0.171±0.009	0.236±0.021	0.079±0.005	0.212±0.010
SCID 17w	0.204±0.012	0.178±0.019	0.248±0.010	0.076±0.006	0.207±0.009
**kidney**					
normal healthy 4w	0.151±0.011	0.112±0.001	0.176±0.011	0.055±0.002	0.134±0.007
normal healthy 17w	0.158±0.009	0.100±0.006	0.205±0.007	0.058±0.007	0.156±0.023
SCID 4w	0.201±0.028	0.129±0.008	0.250±0.017	0.089±0.002	0.196±0.012
SCID 17w	0.204±0.020	0.133±0.003	0.244±0.013	0.089±0.007	0.198±0.018

5. Fatty acid methyl ester levels in RBC membrane

Table S3. Relative percentages (% rel) of fatty acid methyl esters (FAME) from red blood cell (RBC) membrane of normal healthy (Swiss) mice and control SCID mice at different age points (4 weeks and 17 weeks). *p* value represents the comparison between young (4 weeks) and old (17 weeks) mice of each group after conducting unpaired t-test

FAME	normal healthy 4w	normal healthy 17w	*p* value	control SCID 4w	control SCID 17w	*p* value
**14:0**	0.09 ± 0.13	0.61 ± 0.18	0.3270	0.57 ± 0.06	0.55 ± 0.14	0.9075
**15:0**	0.75 ± 0.36	0.56 ± 0.24	0.9054	0.15 ± 0.04	0.15 ± 0.02	>0.9999
**16:0**	28.63 ± 0.75	28.38 ± 0.80	0.9994	35.38 ± 0.46	31.78 ± 0.55	0.0377
**16:1c6**	0.57 ± 0.15	0.27 ± 0.02	0.0713	0.23 ± 0.02	0.23 ± 0.05	>0.9999
**16:1c9**	1.82 ± 0.66	3.09 ± 0.55	0.2269	1.66 ± 0.36	2.89 ± 0.38	0.1426
**17:0**	0.29 ± 0.21	0.43 ± 0.10	>0.9999	0.17 ± 0.00	0.16 ± 0.02	>0.9999
**18:0**	13.15 ± 0.58	10.57 ± 1.01	0.2690	10.73 ± 0.09	12.11 ± 0.47	0.1022
**trans 18:1**	0.22 ± 0.31	nd	0.6568	nd	nd	
**18:1c9**	15.03 ± 0.96	18.06 ± 1.66	0.4361	15.87 ± 0.95	19.09 ± 1.20	0.1701
**18:1c11**	2.67 ± 0.08	3.26 ± 0.03	0.0012	2.70 ± 0.06	2.46 ± 0.07	0.1213
**trans 18:2**	0.26 ± 0.37	nd	0.6611	0.10 ± 0.00	0.10 ± 0.01	0.6985
**18:2 ω-6**	10.63 ± 0.32	12.27 ± 1.28	0.6759	11.22 ± 0.30	11.67 ± 0.25	0.3683
**18:3 ω-6**	0.10 ± 0.14	0.16 ± 0.04	0.9999	0.05 ± 0.05	0.16 ± 0.02	0.1462
**18:3 ω-3**	0.25 ± 0.20	0.24 ± 0.07	0.9286	0.18 ± 0.04	0.18 ± 0.00	0.8995
**20:1c11**	0.54 ± 0.12	0.39 ± 0.02	0.2181	0.48 ± 0.02	0.39 ± 0.04	0.1425
**20:2 ω-6**	0.59 ± 0.09	0.39 ± 0.04	0.2793	0.55 ± 0.02	0.39 ± 0.07	0.1386
**20:3 ω-6**	1.44 ± 0.18	1.35 ± 0.05	0.8947	1.22 ± 0.01	1.14 ± 0.13	0.5880
**trans 20:4**	0.56 ± 0.41	0.20 ± 0.04	0.5000	0.20 ± 0.03	0.24 ± 0.04	0.6513
**20:4 ω-6**	14.50 ± 1.07	13.34 ± 1.51	0.9082	11.53 ± 0.99	10.40 ± 0.95	0.4968
**20:5 ω-3**	0.63 ± 0.04	0.52 ± 0.14	0.7063	0.43 ± 0.03	0.42 ± 0.03	0.9098
**22:5 ω-3**	0.82 ± 0.15	0.70 ± 0.11	0.8803	0.89 ± 0.05	0.67 ± 0.04	0.0663
**22:6 ω-3**	6.46 ± 0.30	5.35 ± 0.48	0.2110	5.70 ± 0.08	4.84 ± 0.30	0.1091

Table S4. Fatty acid families and indices of normal healthy (Swiss) mice at different age points (4 weeks and 17 weeks). *p* value represents the comparison between young (4weeks) and old (17weeks) normal SWISS mice after conducting unpaired *t*-test (n=3).

	**normal healthy 4w**	**normal healthy 17w**	***p* value**
SFA	42.91 ± 1.41	40.55 ± 1.69	0.3594
MUFA	20.63 ± 1.49	25.07 ± 2.18	0.3192
PUFA	35.42 ± 1.15	34.18 ± 0.69	0.9623
PUFA ω-6	27.26 ± 0.97	27.46 ± 0.42	>0.9999
PUFA ω-3	8.17 ± 0.21	6.73 ± 0.53	0.1602
ω-6/ω-3 ratio	3.34 ± 0.07	4.11 ± 0.32	0.6196
SFA/MUFA	2.10 ± 0.22	1.64 ± 0.21	0.7962
total trans	1.56 ± 0.05	0.20 ± 0.04	0.0006
UI	152.47 ± 5.58	146.79 ± 4.57	0.8856
PI	133.73 ± 5.94	119.99 ± 8.77	0.5387

6. Correlation between significantly altered fatty acids and DNA lesions

Table S5. Correlation between the significantly altered fatty acids and DNA lesions using Pearson analysis (n=3). The *p* value of each correlation(r) is presented in parenthesis.

	16:0	18:0	DGLA	ARA	EPA	DHA	5'R-cdA	5'S-cdA	5'R-cdG	5'S-cdG
**16:0**		r = -0.9744 (*p* = 0.1445)	r = 0.0045 (*p* =0.9971)	r = -0.3595 (*p* =0.7659)	r = 0.7534 (*p* =0.4569)	r = 0.6293 (*p* =0.5667)	r = 0.6463 (*p* =0.5527)	r = 0.8429 (*p* =0.3617)	r = -0.6197 (*p* =0.5745)	r = -0.9024 (*p* =0.2836)
**18:0**	r = -0.9744 (*p* =0.1445)		r = -0.2294 (*p* =0.8526)	r = 0.1403 (*p* =0.9104)	r = -0.8820 (*p* =0.3124)	r = -0.7881 (*p* =0.4222)	r = -0.4580 (*p* =0.6972)	r = -0.9423 (*p* =0.2172)	r = 0.4272 (*p* =0.7190)	r = 0.9762 (*p* =0.1391)
**DGLA**	r = 0.0045 (*p* =0.9971)	r = -0.2294 (*p* =0.8526)		r = 0.9315 (*p* =0.2370)	r = 0.6610 (*p* =0.5402)	r = 0.7800 (*p* =0.4304)	r = -0.7602 (*p* =0.4502)	r = 0.5419 (*p* =0.6354)	r = 0.7820 (*p* =0.4284)	r = -0.4350 (*p* =0.7135)
**ARA**	r = -0.3595 (*p* =0.7659)	r = 0.1403 (*p* =0.9104)	r = 0.9315 (*p* =0.2370)		r = 0.3428 (*p* =0.7772)	r = 0.4990 (*p* =0.6674)	r = -0.9444 (*p* =0.2132)	r = 0.1991 (*p* =0.8724)	r = 0.9552 (*p* =0.1914)	r = -0.0777 (*p* =0.9505)
**EPA**	r = 0.7534 (*p* =0.4569)	r = -0.8820 (*p* =0.3124)	r = 0.6610 (*p* =0.5402)	r = 0.3428 (*p* =0.7772)		r = 0.9852 (*p* =0.1099)	r = -0.0150 (*p* =0.9905)	r = 0.9889 (*p* =0.0951)	r = 0.0493 (*p* =0.9686)	r = -0.9632 (*p* =0.1732)
**DHA**	r = 0.6293 (*p* =0.5667)	r = -0.7881 (*p* =0.4222)	r = 0.7800 (*p* =0.4304)	r = 0.4990 (*p* =0.6674)	r = 0.9852 (*p* =0.1099)		r = -0.1864 (*p* =0.8807)	r = 0.9486 (*p* =0.2049)	r = 0.2200 (*p* =0.8588)	r = -0.9028 (*p* =0.2830)
**5'R-cdA**	r = 0.6463 (*p* =0.5527)	r = -0.4580 (*p* =0.6972)	r = -0.7602 (*p* =0.4502)	r = -0.9444 (*p* =0.2132)	r = -0.0150 (*p* =0.9905)	r = -0.1864 (*p* =0.8807)		r = 0.1341 (*p* =0.9144)	r = -0.9994 (*p* =0.0219)	r = -0.2543 (*p* =0.8363)
**5'S-cdA**	r = 0.8429 (*p* =0.3617)	r = -0.9423 (*p* =0.2172)	r = 0.5419 (*p* =0.6354)	r = 0.1991 (*p* =0.8724)	r = 0.9889 (*p* =0.0951)	r = 0.9486 (*p* =0.2049)	r = 0.1341 (*p* =0.9144)		r = -0.0999 (*p* =0.9363)	r = -0.9925 (*p* =0.0781)
**5'R-cdG**	r = -0.6197 (*p* =0.5745)	r = 0.4272 (*p* =0.7190)	r = 0.7820 (*p* =0.4284)	r = 0.9552 (*p* =0.1914)	r = 0.0493 (*p* =0.9686)	r = 0.2200 (*p* =0.8588)	r = -0.9994 (*p* =0.0219)	r = -0.0999 (*p* =0.9363)		r = 0.2209 (*p* =0.8582)
**5'S-cdG**	r = -0.9024 (*p* =0.2836)	r = 0.9762 (*p* =0.1391)	r = -0.4350 (*p* =0.7135)	r = -0.0777 (*p* =0.9505)	r = -0.9632 (*p* =0.1732)	r = -0.9028 (*p* =0.2830)	r = -0.2543 (*p* =0.8363)	r = -0.9925 (*p* =0.0781)	r = 0.2209 (*p* =0.8582)	

Abbreviations: 16:0, palmitic acid; 18:0, stearic acid; ARA, arachidonic acid; cdA, 5',8-cyclo-2'-deoxyadenosine; cdG, 5',8-cyclo-2'-deoxyguanosine; DHA, docosahexaenoic acid; DGLA, dihomo-gamma-linolenic acid; EPA, eicosapentaenoic acid.

7. Tumor size and weight of mice

Table S6. Tumor growth progress through time of tumor-bearing SCID mice

Tumor-bearing SCID	tumor volume (cm)
4w	4.91 x 4.34
4w	5.04 x 4.25
4w	4.84 x 4.71
5w	5.24 x 5.13
5w	5.44 x 4.96
5w	4.72 x 4.06
17w	11.12 x 10.66
17w	13.84 x 12.32
17w	12.06 x 14.89

Table S7. Weight of control SCID mice and tumor-bearing SCID mice

control SCID	(g)
4w	22.6 ± 0.5
17w	26.7 ± 1.8
Tumor-bearing SCID	(g)
4w	21.8 ± 0.5
5w	22.2 ± 0.3
17w	22.0 ± 0.9
